# Mechanisms for Success after Long-term Nutrient Enrichment in a Boreal Forest Understory

**DOI:** 10.1371/journal.pone.0061229

**Published:** 2013-04-03

**Authors:** Tess Nahanni Grainger, Roy Turkington

**Affiliations:** 1 Botany Department and Biodiversity Research Centre, University of British Columbia, Vancouver, British Columbia, Canada; University of Tartu, Estonia

## Abstract

Global levels of reactive nitrogen are predicted to rise in the coming decades as a result of increased deposition from the burning of fossil fuels and the large-scale conversion of nitrogen into a useable form for agriculture. Many plant communities respond strongly to increases in soil nitrogen, particularly in northern ecosystems where nitrogen levels are naturally very low. An experiment in northern Canada that was initiated in 1990 has been investigating the effects of long-term nutrient enrichment (fertilizer added annually) on a boreal forest understory community. We used this experiment to investigate why some species increase in abundance under nutrient enrichment whereas others decline. We focused on four species that differed in their responses to fertilization: *Mertensia paniculata* and *Epilobium angustifolium* increased in abundance, *Achillea millefolium* remained relatively constant and *Festuca altaica* declined. We hypothesized that the two species that were successful in the new high-nutrient, light-limited environment would be taller, have higher specific leaf area, change phenology by growing earlier in the season and be more morphologically plastic than their less successful counterparts. We compared plant height, specific leaf area, growth spurt date and allocation to leaves in plants grown in control and fertilized plots. We demonstrated that each of the two species that came to dominate fertilized plots has a different combination of traits and responses that likely gave them a competitive advantage; *M. paniculata* has the highest specific leaf area of the four species whereas *E. angustifolium* is tallest and exhibits morphological plasticity when fertilized by increasing biomass allocation to leaves. These results indicate that rather than one strategy determining success when nutrients become available, a variety of traits and responses may contribute to a species' ability to persist in a nutrient-enriched boreal forest understory.

## Introduction

Atmospheric deposition of nitrogen from the burning of fossil fuels, inputs from industrial fertilizers and faster mineralization rates resulting from rising temperatures are predicted to dramatically increase levels of available nitrogen in the coming decades [1]. As nitrogen enrichment becomes one of the most pressing environmental concerns facing ecosystems worldwide, it is imperative to understand how elevated levels of this nutrient will alter vegetation composition and dynamics. Experimental evidence from a variety of ecosystems including grasslands, temperate forests and arctic tundra has shown that nutrient enrichment leads to species loss when some species are out-competed to local extinction in the altered environment [2]–[6]. Classic theory, along with recent experimental evidence, provides several potential mechanisms for the observed variability in species' success under altered nutrient regimes.

Grime [7] and Chapin [8] describe several characteristics that affect a plant's ability to utilize above and below-ground resources and are indicative of a species' relative success under high and low nutrient conditions. When nutrients are added to vegetation, biomass production increases and light becomes the new limiting resource, and the species that thrive tend to be those that have thin leaves and grow faster and taller (acquisitive strategy) [7], [8]. In contrast, species that are slower-growing, shorter and have thicker leaves require less nutrients and tend to succeed under nutrient-limited conditions (conservative strategy) [7], [8]. Recently, there has been a focus on specific functional traits as quantifiable indicators of plant strategies [9]–[11]. When functional trait theory is applied to experiments investigating the effects of nutrient addition on plant communities, Grime and Chapin's theories are corroborated; traits favouring maximum light capture including high specific leaf area (SLA), tall stature and fast growth rates are more prevalent in fertilized communities [12]–[14]. Likewise, phenology, and particularly the timing of early-season growth, influences a species' ability to access light at a critical developmental period early in the growing season and could further dictate whether a species is successful in nutrient-enriched conditions [12], [15].

Harper [16] introduced plant ecologists to the concept of variable allocation to different tissues as a strategy to cope with changing environmental conditions. Tilman [17], [18] and Bazzaz [19] subsequently proposed theoretical frameworks relating changes in resource availability to altered allocation of biomass to root, stem, leaf and reproductive tissues. More recent experimental evidence indicates that when environmental conditions change, species vary in their ability to modify allocation to above-ground tissue types such as stems and leaves [20]–[22]. Changing allocation patterns can be an important strategy for coping with altered resource levels, and species able to so may gain a competitive advantage [17]–[19]. However, caution is needed when considering how nutrient addition affects allocation to tissue types; because there can be an inherent relationship between plant size and allocation to certain tissues, observed differences in the amount of biomass allocated to a tissue between resource treatments could simply be a result of plants being larger when fertilized [23], [24]. Consequently, it is more informative to examine how the relationship between allocation to a tissue and total plant size is altered by nutrient addition (allometric approach) than to simply test for differences in the ratios of various tissues [23], [25], [26]. In this way, it is possible to distinguish between apparent plasticity (due to size differences) and true plasticity (due to treatment effects) [21], [25]. If the ability to be morphologically plastic in a changing environment gives some species an advantage under nutrient-enriched conditions, then carefully evaluating how fertilizer-induced plasticity varies by species may provide insight into the variable success of species in nutrient-enriched plant communities [20], [22], [27].

Traits and responses that determine which species are successful under nutrient enrichment are context-dependent and vary greatly by ecosystem, so conclusions derived in one ecosystem cannot necessarily be applied to others [2], [28]. It is thus necessary to consider vegetation responses to increased nutrients in an ecosystem-specific context. However, most of the research investigating changes in community composition after fertilization has been conducted in grasslands [13], [14], [28]–[30]. To increase the generality and applicability of the current models and their predictions, more research is required on the importance of various strategies in determining success in nutrient-enriched non-grassland ecosystems.

If plant community dynamics under the nitrogen-enriched conditions that are predicted for the coming decades mirror responses detected in fertilizer experiments, species loss could be widespread [2]–[5]. The effect of nitrogen enrichment could be most notable in the arctic and sub-arctic, where temperature increases are expected to be especially pronounced and vegetation is adapted to very low soil nutrient levels [6]. It is therefore essential to increase our understanding of how nitrogen enrichment affects vegetation in these northern ecosystems. A long-term experiment in the boreal forest of northern Canada provided an opportunity to test the importance of functional traits and morphological plasticity in determining success under an altered nutrient regime. The experiment was set up in 1990 to investigate bottom up (nutrient addition) vs. top-down (grazing by herbivores) controls on the understory vegetation [5], [31]. When data for the present study were collected, plots had been fenced to exclude herbivores and/or fertilized to add macronutrients for 22 years. Over these 22 years of treatment, marked changes in vegetation composition were observed in fertilized plots [5], [32]. While some species increased in abundance in nutrient-enriched conditions, others declined to local extinction, leading to a decline in species richness in fertilized plots [5]. In contrast, fencing had almost no effect on this plant community. For this reason, we have concentrated primarily on the effects of nutrient enrichment, although all parameters measured were also tested for fencing effects.

In this study, we focus on four understory species that had varying levels of long-term success in fertilized conditions. Although they all initially increased in abundance in fertilized plots, more than two decades of treatment eventually resulted in very different levels of success for these focal species; bluebells (*Mertensia paniculata* (Aiton) G. Don) and fireweed (*Epilobium angustifolium* L. s.l.) came to dominate fertilized areas while yarrow (*Achillea millefolium* L. var. *borealis* (Bong) Farwell) stabilized to abundances similar to unfertilized plots and northern rough fescue (*Festuca altaica* Torr.) declined to near elimination under nutrient enrichment. We test the hypothesis that the most successful species (those that increased in abundance) in fertilized conditions over the long term are those that:

- have traits that are beneficial in light-limited conditions, including tall stature, growth early in the season, and high specific leaf area.
- are morphologically plastic and increase biomass investment to leaves when fertilized.

## Methods

### Study Site

The study site is in the boreal forest near Kluane Lake in southwestern Yukon Territory (138°15'W 60°59'N), and is described in detail by Turkington et al. [5]. All necessary permits were obtained from the Government of Yukon prior to the beginning of the experiment. The area receives an annual mean precipitation of ca. 290 mm and daily mean temperatures range from 4.7°C–18.0°C during the June–August growing season [33]. The forest is moderately open (45–60% canopy cover, 160–220 stems/ha) and is dominated by white spruce (*Picea glauca* (Moench) Voss) interspersed with stands of trembling aspen (*Populus tremuloides* Michx.) and balsam poplar (*Populus balsamifera* L.). In 1995 an outbreak of spruce bark beetle killed off many trees in this area, allowing for more penetration of light into the understory. There is a well-developed shrub layer consisting primarily of willows (*Salix glauca* (L.), dwarf birch (*Betula glandulosa* Michx.) and soapberry (*Shepherdia canadensis* (L.) Nutt.). In addition to the four focal understory species used in this study, arctic lupine (*Lupinus arcticus* S. Wats.), twin-flower (*Linnaea borealis* L.) and bearberry (*Arctostaphylos uva-ursi* (L.) Spreng.) are also common at this site. The snowshoe hare (*Lepus americanus* Erxleben) is the main herbivore in the system, and experiences an 8–12 year population cycle [34]. In 2011 when the current study was conducted, snowshoe hares were at a low point in their cycle with a density of 10 hares/km^2^ [35].

### Experimental Design

Experimental plots were set up in 1990. Fertilizer was applied to increase soil nutrients and fences were erected to exclude snowshoe hares. There are sixteen 5 m×5 m plots with fully crossed fertilizer and fencing (exclosure) treatments (+/0 fertilizer, +/0 fencing) for a total of four treatments. Experimental plots were placed in semi-open areas within the understory with few shrubs and no rooted trees, and treatments were randomly assigned to each plot for a total of four replicates of each treatment. Since 1990, granular fertilizer (N:P:K 35∶10∶5) has been applied to all fertilized plots each year shortly after snow melt, between mid-May and early June. Fertilizer was applied at a rate of 1.25 kg per 5 m×5 m plot per year, resulting in a total addition of 17.5 g N/m^2^/year, 5 g P/m^2^/year and 2.5 g K/m^2^/year, which is a fertilization rate consistent with other long-term fertilization experiments [36], [37]. Natural mineralization rates of nitrogen in this region are approximately 4.7 g/m^2^/year [38]. After ten years, treatment was stopped (fertilizer stopped or fences removed) in half of each plot to assess the potential for the vegetation to recover. We used only the half of each plot that was treated for the full 22 years, so each plot was 5 m×2.5 m.

### Study Species

Four common understory species were selected for this study: *M. paniculata*, *E. angustifolium*, *F. altaica* and *A. millefolium var. borealis*. These species are all native herbaceous perennials. These species were selected because they span the range from successful to very unsuccessful in fertilized plots. *Achillea millefolium* and *M. paniculata* have two distinct growth forms: a rosette form consisting of several leaves and no stem (not sexually reproductive), and an erect form (sexually reproductive). *Epilobium angustifolium* has only an erect form, and flower parts, although often stunted, are almost always present. *Mertensia paniculata*, *E. angustifolium* and *A. millefolium* are all capable of reproducing asexually though the underground spread of rhizomes, whereas *F. altaica* is a densely-tufted bunchgrass that grows from fibrous roots.

### Abundance

Treatment effects on abundance of focal species were measured by recording the percent cover of each species in all plots during surveys conducted between July 11^th^ and 13^th^ 2011. In all plots, a sampling pin was dropped every 10 cm along five 2 m long transects and each of the focal species was recorded as present or absent at each pin drop location. Percentage cover for each species was the percentage of points at which that species was present within each plot.

### Traits

Five individuals of each growth form (reproductive and non-reproductive) of E. angustifolium and M. paniculata were randomly selected from each plot to measure height and specific leaf area (SLA). Because reproductive *F. altaica* and *A. millefolium* were virtually absent in most plots, only non-reproductive (rosette form) individuals of these species were sampled. To avoid edge effects, plants were not sampled within 20 cm of the edge of the plot. Plants were also excluded if they were within 15 cm of a previously selected individual of the same species to minimize the chances of selecting two individuals of the same clone. Sampling was conducted during the period of peak flowering for each species. *Mertensia paniculata* was sampled on June 26^th^, *F. altaica* on July 6^th^ and *A. millefolium* on July 19^th^. Because fertilization advances the phenology of *E. angustifolium* by seven days in these plots [39], *E. angustifolium* was sampled in fertilized plots on August 1^st^ and in unfertilized plots on August 8^th^.

#### Height

The height of each selected individual was recorded on live plants just prior to harvesting.

#### Specific Leaf Area

Leaves were collected from each selected individual to calculate SLA. To consistently collect young, fully expanded leaves at approximately the same location on all *M. paniculata* and *E. angustifolium* individuals, we selected the first two fully expanded leaves just below the lowest flower. For those plants with no erect stem (*F. altaica* and *A. millefolium*), the longest leaf was collected for SLA measurements. Any leaves exhibiting obvious herbivory or pathogen damage were excluded, and in those rare cases, the next leaf down on the stem or the next longest leaf was used. Harvested leaves were immediately placed between damp paper towels and transported to the lab. Leaves were re-wetted upon return to the lab, placed between wet paper towels in a cooler and left for 18 hours in a refrigerator (4°C) for re-hydration [40]. Leaves were re-wetted every six hours during this re-hydration period. Re-hydrated leaves were patted dry, weighed and scanned to obtain leaf area using ImageJ software [41]. Due to the fineness and rolled morphology of *F. altaica* leaves, it was not possible to obtaining an accurate leaf area using a scanner. Therefore 20 cm sections were cut at the centre of all *F. altaica* leaves and the width of the unrolled leaf was measured at its centre. Assuming a roughly uniform width for the 20 cm centre section of the leaf, the area of the leaf section was calculated. Although it was also difficult to obtain an accurate leaf area for *A. millefolium* due to their finely divided morphology, scanned images of carefully positioned leaves provided a reliable estimate of leaf surface area. After scanning, all leaves were dried for 48 hours at 60°C and reweighed. SLA was calculated as the area per unit dry mass of each leaf.

#### Growth Spurt Date

The effect of fertilization on timing of growth of each species was quantified using “growth spurt date,” which was defined as the date at which plants in fertilized plots were significantly taller than their unfertilized counterparts. At the beginning of the growing season, just after snowmelt (June 3^rd^ 2011), five emerging individuals of each species were randomly selected in each of the sixteen plots; these were different individuals from those used in height, SLA and biomass measurements. For *F. altaica*, an individual was a ramet within the larger clump, identified by the sheath at the base of the ramet. Target individuals were tagged using an aluminum tag with a unique ID number attached to the base of the plant. Every third day between June 3^rd^ and July 24^th^ (18 surveys) the heights of all selected individuals were recorded.

#### Biomass Allocation to Leaves

Because non-reproductive *A. millefolium* and *F. altaica* have a rosette morphology with only one tissue type (leaf), only reproductive *E. angustifolium* and *M. paniculata* (the same five individuals per plot collected for trait data) were used for biomass measurements. Each individual was harvested, divided into leaves, stems and flowers, oven-dried at 60°C for 48 hours and weighed.

### Data Analysis

Species were analyzed separately except when testing for intraspecific differences in height and SLA of unfertilized plants (species were compared using pairwise comparisons and Bonferroni's correction). Analyses of traits and biomass allocation were based on mean values of individuals in a plot. Some plots did not contain five individuals of one or several species, and those plots containing fewer than three individuals of a species were excluded from analysis. Two-way ANOVAs were used to test for effects of fertilizer and exclosures (fencing) treatment on height, percent cover and SLA. Percent cover data were rank transformed to meet model assumptions. Fertilizer and fencing effects on biomass allocation to leaves and stems in *M. paniculata* and *E. angustifolium* were assessed using two separate ANCOVAs (one for fertilizer effects and one for fencing effects) with total plant biomass used as a covariate [21], [25]. Two-way ANOVAs were performed on height of each species at each date, and the first date at which fertilized plants were significantly taller than unfertilized plants was the growth spurt date. Repeated measures ANOVAs were performed using the SPSS statistical package [42] and all other data analyses were performed using the R statistical package [43].

## Results

### Abundance

**Figure 1:**
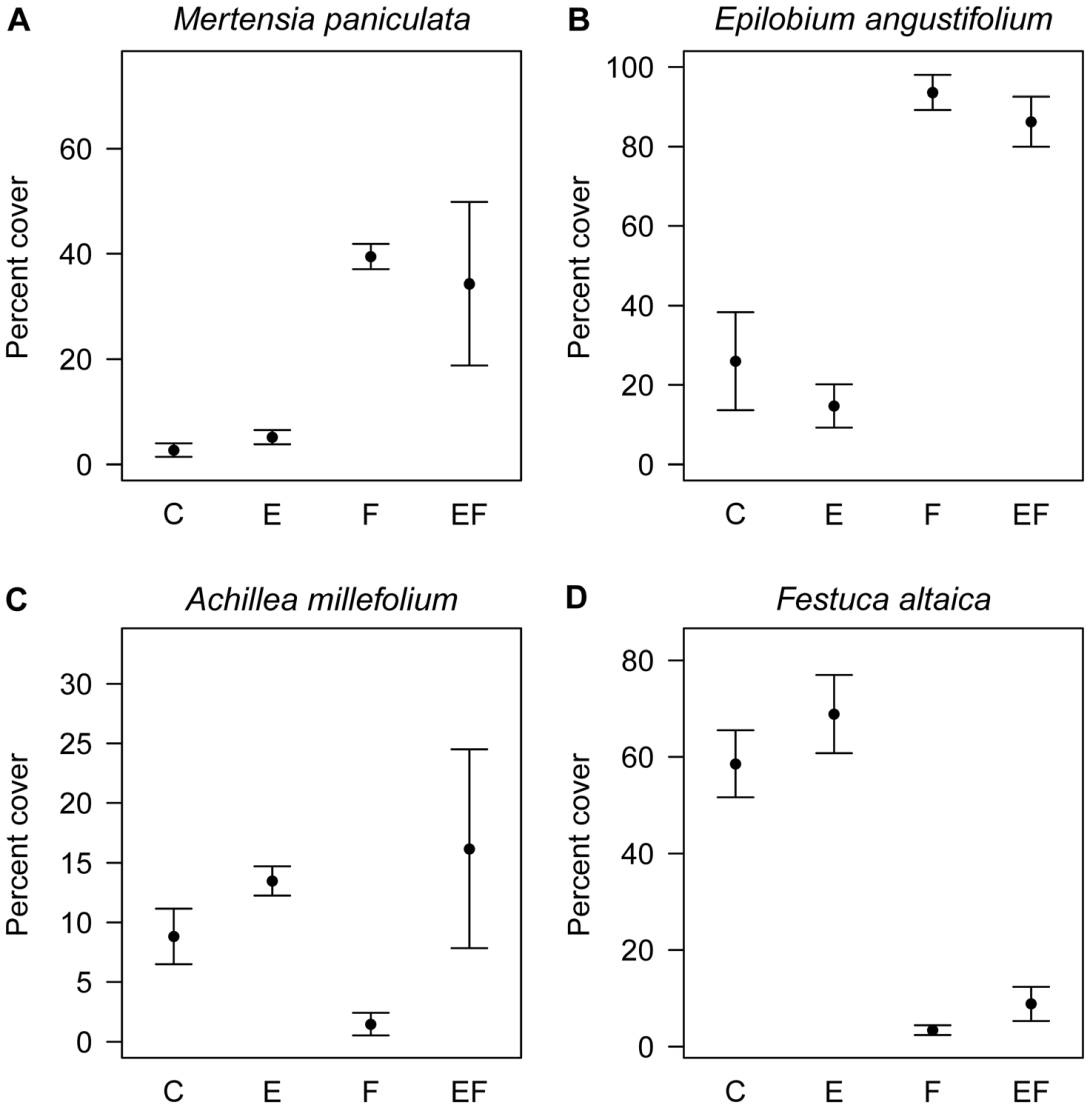
Percent cover of four focal species in all treatments. Percent cover (±1 SE) of four boreal forest understory species in control (C), exclosure (i.e. fencing) (E), fertilized (F) and fertilized and exclosure (FE) plots. Points represent averages from four plots per treatment. Note the different scales on the Y-axes.

Fertilization increased cover of *M. paniculata* and *E. angustifolium* and decreased cover of *F. altaica*, while fencing increased cover of *A. millefolium* (Table 1; Figure 1). There was no interaction between fertilizer and fencing treatments for any species.

**Table 1:** Summary of ANOVAs testing for effects of fertilizer and fencing treatments on percent cover of four boreal forest understory species.

		Fertilizer		Fencing		Interaction	
Species	d.f.	F	p	F	p	F	p
*Mertensia paniculata*	1,12	12.06	**0.005**	0.25	0.629	2.89	0.115
*Epilobium angustifolium*	1,12	41.80	**<0.001**	1.47	0.249	0.16	0.693
*Achillea millefolium*	1,12	3.58	0.083	4.87	**0.048**	0.90	0.363
*Festuca altaica*	1,12	40.76	**<0.001**	1.20	0.294	0.16	0.693

Bold values are significant at p<0.05.

### Traits

**Figure 2:**
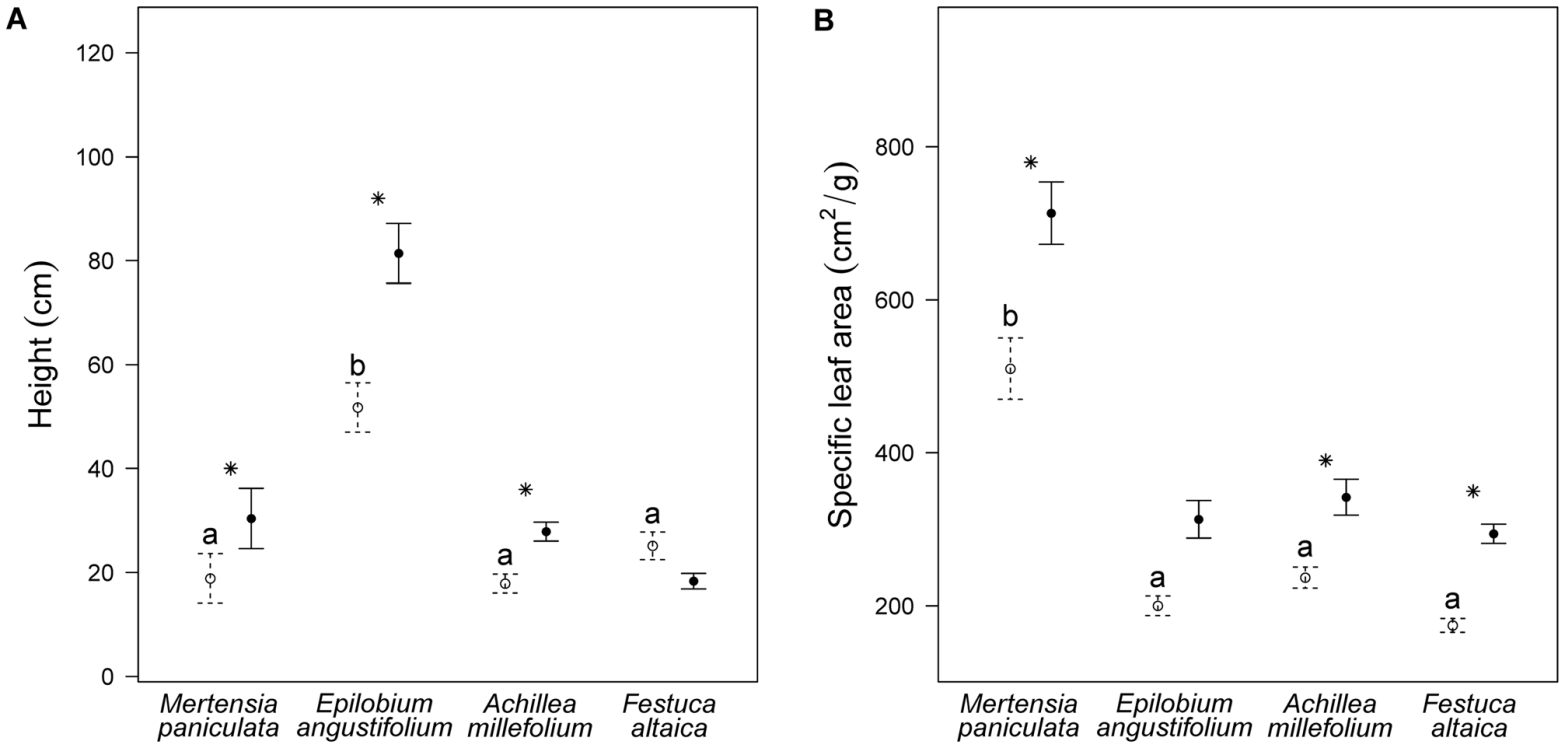
Height and specific leaf area of four focal species in fertilized and unfertilized plots. Height (±1 SE) and specific leaf area (±1 SE) of four boreal forest understory species grown in fertilized (solid lines) and unfertilized (dashed lines) conditions. Unfertilized plants include those in control plots and fenced plots, and fertilized plants include those in fertilized plots and fenced + fertilized plots. Asterisks indicate a significant difference between unfertilized and fertilized treatments, within a species. Species with the same letter are not significantly different (p<0.05) using pairwise comparison of species' means for unfertilized plants. Data for each species are from five individuals in each of eight unfertilized plots.

In unfertilized plots, *E. angustifolium* was the tallest species and *M. paniculata* had the highest SLA (Figure 2). Fertilization caused an increase in SLA for *M. paniculata*, *A. millefolium* and *F. altaica*, and an increase in height for *M. paniculata*, *E. angustifolium* and *A. millefolium* (Figure 2). *Achillea millefolium* also had a higher SLA in fenced plots (Figure 2b).

**Figure 3:**
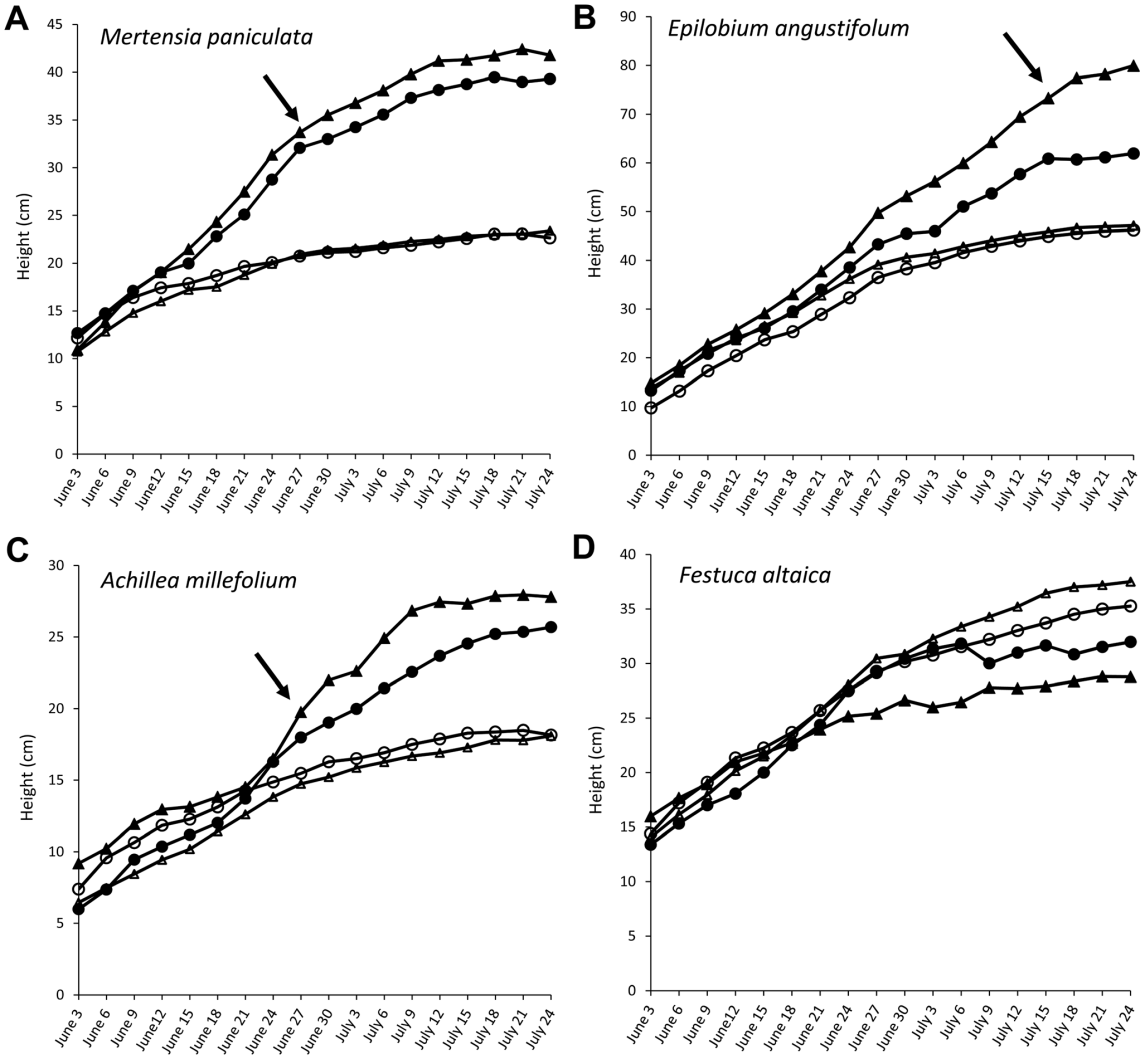
Heights of four focal species in all treatments throughout growing season. Heights of five individuals in each plot were measured every third day between June 3^rd^ and July 24^th^ 2012. Solid symbols show the average height of plants grown in fertilized treatments, open symbols are unfertilized plants. Triangles show fenced treatments, circles are unfenced. Arrows indicate, for each species, the date at which plants in fertilized treatments were significantly (p<0.05) taller than plants in unfertilized treatments.

*Mertensia paniculata* and *A. millefolium* had growth spurts on June 27^th^ and *E. angustifolium* had its growth spurt on July 15^th^ (Figure 3). *Festuca altaica* did not have a growth spurt date because there was no date at which fertilized plants were significantly taller than unfertilized plants for this species.

### Biomass Allocation to Leaves

**Figure 4:**
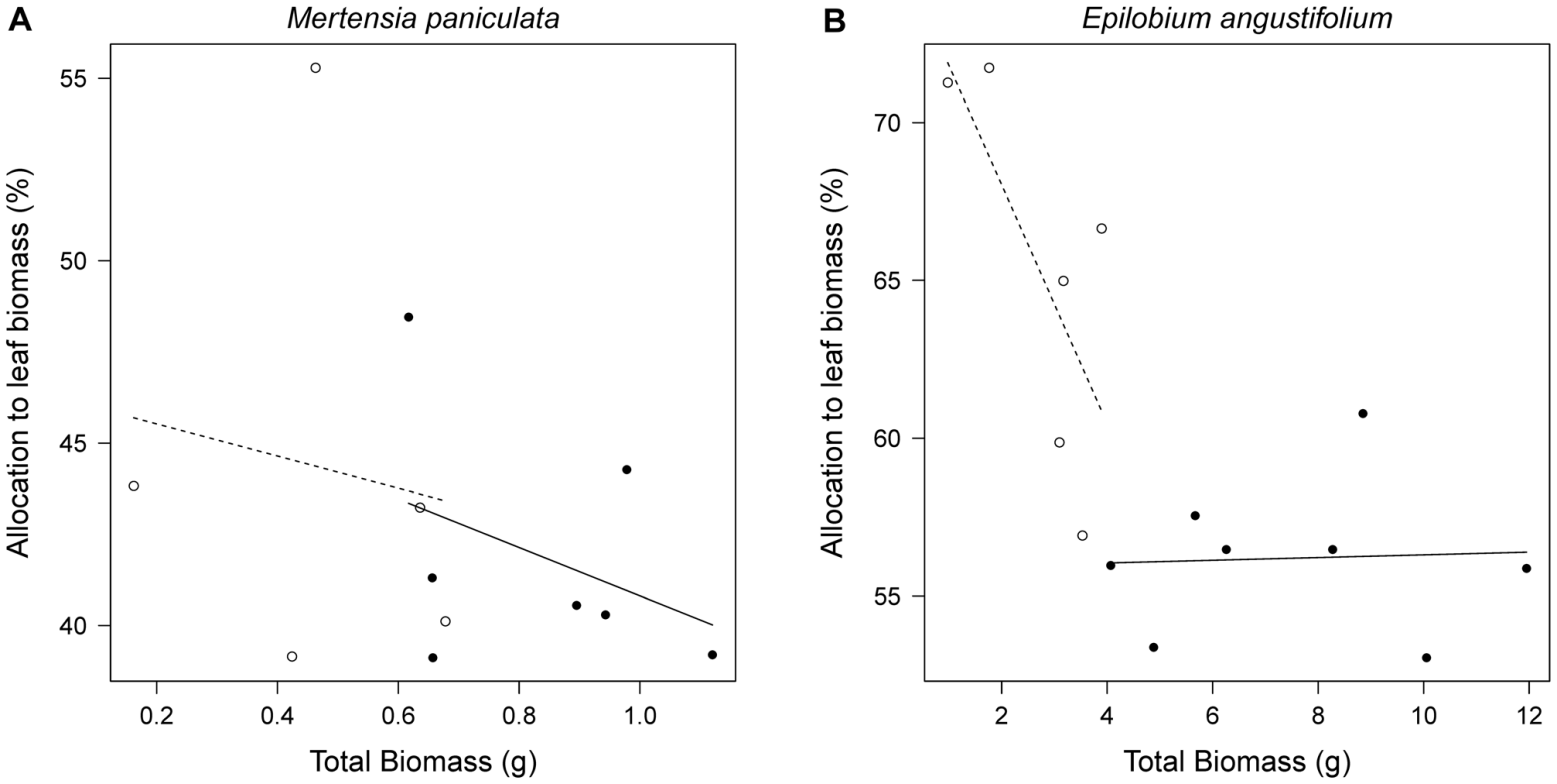
Allocation of biomass to leaves of *Mertensia paniculata* and *Epilobium angustifolium* in fertilized and unfertilized plots. The relationship between proportional allocation to leaves (leaf biomass/total biomass) and total plant biomass for (a) *M. paniculata* and (b) *E. angustifolium* in fertilized (closed symbols, solid lines) and unfertilized plots (open symbols, dashed lines). Unfertilized plants include those in control plots and fenced plots, and fertilized plants include those in fertilized plots and fenced + fertilized plots. Each point represents a plot average for five individual plants.

There was a significant relationship between allocation to stems and total plant biomass for *M. paniculata* (proportional allocation to stems increased with plant size), however fertilization had no effect on biomass allocation for this species (Table 2; Figure 4a). *Epilobium angustifolium* showed a negative relationship between proportional allocation to leaves and total plant biomass, and the reverse trend for allocation to stems, so larger plants had proportionally less biomass in leaves and more in stems (Table 2). Therefore, fertilized *E. angustifolium*, which were larger than their unfertilized counterparts, had lower proportional biomass allocated to leaves as a result of the inherent relationship between plant size and leaf allocation for this species (Figure 4b). This was due to an inherent size-allocation relationship, rather than a treatment effect. However, because fertilizer weakened this negative relationship between allocation to leaves and total plant size, large fertilized plants invested more biomass in leaves than they would have had they not been fertilized. There was no effect of fencing on proportional allocation of biomass to leaves or stems for *E. angustifolium* or *M. paniculata* (Table 2).

**Table 2:** Summary of ANCOVA testing effects of fertilizer treatment on the proportional allocation to leaves and stems of *Mertensia paniculata* and *Epilobium angustifolium* with total plant biomass used as a covariate.

			Total Biomass^1^		Fertilizer^2^		Interaction^3^	
Species	Trait	d.f.	F	p	F	p	F	p
*Mertensia paniculata*	Leaves	1,8	1.08	0.328	0.01	0.936	0.02	0.899
	Stems	1,8	375.14	**<0.001**	0.03	0.873	0.03	0.866
*Epilobium angustifolium*	Leaves	1,10	16.28	**0.002**	6.05	**0.033**	6.40	**0.030**
	Stems	1,10	9.95	**0.010**	1.54	0.243	4.78	0.054

Bold values are significant at p<0.05. ^1^Total Biomass refers to whether there is a significant relationship between total plant biomass and proportional allocation to stems or leaves. ^2^Fertilizer refers to effects of fertilizer treatment on proportional allocation to stems or leaves with total plant biomass as a covariate. ^3^Interaction refers to whether fertilizer treatment changes the relationship between total plant biomass and proportional allocation to stems or leaves.

## Discussion

After 22 years of treatment, release from nutrient limitation has substantially altered the vegetation dynamics in fertilized plots, whereas fencing had virtually no effect on this plant community. An annual input of nutrients increased biomass production, and the subsequent crowding combined with the dominance of two tall species has led to fertilized plots having a more light-limited environment than control plots [32], [44]. The major changes in species abundances after 22 years of fertilization and the observed loss of species in fertilized plots are most likely due to a change in the above-ground competitive environment that favours species better able to capture light [12], [30], [45], [46]. Here we show that the two most successful species in fertilized plots, *M. paniculata* and *E. angustifolium*, each had a different combination of traits and responses that conferred success in the new light-limited environment.

*Epilobium angustifolium* demonstrated two strategies that are known to be advantageous in light-limited conditions. Firstly, it was the tallest of the four species under control conditions and grew even taller in fertilized plots, which likely contributed to its competitive dominance when fertilized [12], [47]. Secondly, this species demonstrated morphological plasticity by investing more biomass into leaves when fertilized. Increased allocation to leaves in nutrient-rich, light-limited conditions is a response adopted by many plant species to optimize light capture, and is a recognized characteristic of acquisitive species [20]–[22], [25]. In contrast, the other species that demonstrated long-term success in these plots, *M. paniculata*, was not taller than its neighbours and did not increase investment to leaves when fertilized. However, under control conditions *M. paniculata* had larger, thinner leaves (higher SLA) than any other species, and these leaves became even thinner when fertilized. High SLA has been associated with an acquisitive life history strategy adapted to high nutrient, light-limited conditions [11], [13], [48]. *M. paniculata* also emerges early in the growing season [39]. This species was therefore predicted to have the earliest growth spurt date; however fertilized plants were not significantly taller than their unfertilized counterparts until June 27^th^ (24 days into the 52 day monitoring period). Heights of fertilized and unfertilized *A. millefolium* also diverged on June 27^th^ and *E. angustifolium* diverged on July 15^th^. Although it was hypothesized that an early-season growth spurt induced by fertilization could give some species an advantage over their neighbours, there was no evidence that growth spurt date was related to success in fertilized plots. 

Theory predicts a switch from dominance by species with a conservative strategy to those with an acquisitive strategy when nutrients are added [7], [8] and this has been observed experimentally [4], [12], [49]. Although neither of the two successful species had all the traits and responses that we predicted would help them succeed under nutrient enrichment, each had at least one acquisitive trait. These results indicated that in this boreal forest understory, different combinations of traits and responses, rather than a single best strategy, have helped two species dominate in nutrient-enriched conditions.

Despite *A. millefolium* growing taller and both *A. millefolium* and *F. altaica* having higher SLA in fertilized plots, these species were unable to contend with the light capturing abilities conferred by the much taller stature and higher SLA of their more competitive neighbours. Although a scarcity of erect, reproductive *A. millefolium* and *F. altaica* precluded analysis of morphological plasticity in these species, the presence of some tall reproductive *A. millefolium* individuals exclusively in fertilized plots (unpublished data) may have been a response to the light-limited conditions created by tall neighbours. This could have aided *A. millefolium* in avoiding elimination from fertilized plots. *Festuca altaica*, in contrast, declined to near local extinction in fertilized plots. The result contrasts with other studies that describe graminoids as one of the more successful functional groups in fertilization experiments [4], [28], [50]–[52]. However, these studies were conducted in grasslands rather than forested ecosystems (but see [53], [54]), where graminoids are often the tallest species present and light is less limiting. As well, in this ecosystem the bunched growth form of *F. altaica* contrasts with the rhizomatous form of most of its neighbours. This could have further contributed to this species failure to thrive in fertilized plots [47], although some research has shown little difference in the success of clumped and rhizomatous species under nutrient-enrichment [4]. Finally, results of many of the studies that detected an increase in grasses were reported after a much shorter treatment period (often 1–5 years) than we report here. For example, Nams et al. [55] reported an increase in *F. altaica* abundance in a 2-year study in the same region. Even during the first ten years of the experimental plots used in the current study, both *F. altaica* and *A. millefolium* increased in abundance in fertilized plots. Only in years 10–15 when *E. angustifolium* and *M. paniculata* began to dominate the fertilized plots did *F. altaica* and *A. millefolium* decline [5], [32]. Such long-term shifts highlight the need for multi-decadal experiments in order to detect trends in vegetation dynamics that may not be evident over only a few years.

Several other herbaceous species not examined in this study were almost entirely eliminated from fertilized plots in the first ten years of the study [5]. These species were mostly short, slow-growing woody species with low SLA (conservative strategy) such as *Arctostaphylos uva-ursi* and *Linnaea borealis* [5]. Although these species' responses to fertilizer could not be measured due to lack of replicates in fertilized plots, the competitive exclusion of species with a conservative strategy supports the conclusion that only the more acquisitive species that are able to compete in a light-limited environment by having a high SLA and tall stature will be competitive when nitrogen becomes readily available [7].

### Lack of Fencing Effects

In contrast to the notable effects of fertilization on this plant community, fencing had almost no effect on the studied species. Herbivore exclusion likely had little effect because snowshoe hares were at very low levels when data for this study were collected [35]. In addition, feeding trials conducted in this region showed that the summer diet of snowshoe hares consists mainly of woody shrubs (*Salix spp*., *Shepherdia canadensis* and *Betula glandulosa*) and *F. altaica* [56]. As such, herbivore exclusion had little effect on any of the plant species; the few fencing effects that were detected were only marginally significant; *Achillea millefolium* was slightly more abundant (p = 0.048) and had slightly higher SLA in fenced plots (p = 0.042). These results were likely caused by the presence of two fenced and fertilized plots in which *A. millefolium* was very successful and abundant.

### Future Directions

Although the nitrogen level applied in this experiment is comparable to treatments used in other fertilization experiments [36], [37], these application rates are much higher than natural deposition rates predicted for this region [1]. The present estimate for critical nitrogen loads for the boreal forest (the level below which this ecosystem will not experience harmful effects) is between 0.6 g and 1.5 N/m^2^/year, in contrast to the 17.5 g N/m^2^/year applied in this study [54]. The high rate of application used here is justified in experimental approaches to determine whether a system will respond to treatment at any level. Having detected major responses at this high level of application, it would be beneficial in future studies to measure responses to nitrogen applied at a level that more realistically approximates future conditions. It would also be informative to examine changes in allocation to root tissue as a result of fertilizer treatment. It is likely that some species are better able to divert resources away from roots and allocate biomass to above-ground growth when released from nitrogen limitation, which would give these species an advantage [8]. Unfortunately, the experimental plots used in this study continue to be treated and monitored, and destructive harvesting of roots would disrupt the long-term integrity of this experiment.

## Conclusion

A recent focus on plant traits and their usefulness in assessing the causes and consequences of ecosystem change has led to several studies investigating which traits determine species success in high nutrient conditions [4], [13], [14]. A separate body of work has investigated how a species' ability to alter allocation to different tissue relates to its ability to compete when released from nutrient limitation [20], [22], [27]. Rarely have both mechanisms for success been considered in tandem. Our study shows that in a nitrogen-enriched boreal forest understory, successful strategies are those that confer an advantage in a light-limited environment: tall stature, high SLA and morphological plasticity. Species that lacked these traits and responses were unable to compete. In addition, we demonstrated that competitive dominance is not achieved by a single best-strategy. Rather, successful species had different combinations of traits and responses that allowed them to thrive in nutrient-enriched conditions.
